# Providing reproductive health services for women who inject drugs: a pilot program

**DOI:** 10.1186/s12954-020-00395-y

**Published:** 2020-07-14

**Authors:** Lauren Owens, Kelly Gilmore, Mishka Terplan, Sarah Prager, Elizabeth Micks

**Affiliations:** 1 Department of Obstetrics and Gynecology, University of Michigan|, Ann Arbor, USA; 2 Department of Obstetrics and Gynecology, University of Washington, Seattle, USA; 3 Department of Obstetrics and Gynecology, Department of Psychiatry, Virginia Commonwealth University, Richmond, USA

**Keywords:** Reproductive health, Needle syringe programs, Syringe exchange programs, Substance use disorder

## Abstract

**Background:**

Needle syringe programs (NSPs), a proven harm reduction strategy for people who inject drugs, frequently offer limited healthcare services for their clients. Women who inject drugs face multiple barriers to accessing reproductive health care in traditional settings: personal histories of trauma, judgmental treatment from providers, and competing demands on their time. Our aim was to implement patient-centered reproductive healthcare services at a Seattle NSP.

**Methods:**

We interviewed clients and staff of an NSP in Seattle and staff of other community-based organizations serving women who inject drugs, then used the Consolidated Framework for Implementation Research to code transcripts deductively. Based on our qualitative work, we implemented reproductive health care at the NSP program 1 day per week. We evaluated the implementation by surveying staff and clients and auditing charts over a 9-month period.

**Results:**

Clients and staff (*N* = 15 for clients, *N* = 13 for staff) noted a high unmet need for trauma-informed, accessible reproductive health care. We successfully implemented reproductive health care services including short- and long-acting contraception, sexually transmitted disease testing, and cervical cancer screening. Survey data was limited but demonstrated client satisfaction with services.

**Conclusions:**

Integrating reproductive health care into an NSP’s clinical services is feasible and can be a source of low-barrier preventive care for women unable to seek gynecologic care elsewhere.

## Background

A 2014 study estimated that over 0.3% of Americans 13 years of age and older, representing 750,000 people, injected drugs in the preceding year [1]. In King County, the county containing Seattle, approximately 20,000 people (1.3% of adults aged 18 and older) inject drugs [2, 3]. Many of these people benefit from local needle syringe programs (NSPs). NSPs are a proven harm reduction strategy that can decrease the spread of infectious diseases [4]. The majority of NSPs in the United States provide clinical services such as testing for HIV and other sexually transmitted diseases (STDs) [5]. Nearly all NSPs provide condoms to clients as part of their harm reduction mission, but data on broader contraceptive provision are limited [5].

Women who use drugs are twice as likely to have unintended pregnancies as women in the general population. In a study of 302 women in treatment for substance use disorder, nearly 8 of 10 pregnancies in the year prior to the study were unintended [6]. While women in substance use disorder treatment use highly effective contraceptives at rates lower than the general population, little is known about contraceptive usage rates among women who inject drugs who are not in drug treatment [7]. A pilot study involving 152 clients over approximately 2 years demonstrated the feasibility of providing certain methods of contraception (the pill, patch, injection, vaginal ring) at a mobile syringe exchange in Baltimore [8]. However, this project did not include more effective methods such as intrauterine devices (IUDs) and implants.

In addition to other barriers in accessing healthcare services, people who inject drugs (PWID) experience discrimination and stigma that are independently associated with poor health outcomes [9, 10]. One cross-sectional study compared 224 PWID to other people who do (*N* = 463) and do not (*N* = 468) use drugs; this study found that people who inject drugs were less likely to have used primary care services than others [11]. A second study of 536 people found that PWID were more likely than other people who used drugs and people who did not use drugs to not receive needed health care [12]. Although these studies did not explore the relationship between criminalization and prohibition of injection drug use in PWID’s inability to access needed care, both factors are likely contributors. Lack of insurance, a reflection of structural inequities that may more harshly impact PWID, was associated with not receiving care. A cross-sectional study of 235 women, including 93 women who used drugs, found that women who used drugs were significantly less likely to seek needed healthcare and more likely to rely on the emergency department for their care compared to those who did not use drugs [13].

Trusted healthcare and social service providers create positive experiences for PWID that can increase their usage of social and health services [14, 15]. Needle syringe programs are one place where PWID report feeling safe and comfortable seeking a variety of services [10]. This may be due to the fact that NSPs operate under a harm reduction model, accepting their clients and their substance use disorders without expectation of change [15]. The Lancet’s report on sexual and reproductive health (RH) rights notes the need for “dedicated services for those who have no access.” [16] Although the report does not specifically list PWID when making this recommendation, the need for accessible sexual and RH care for PWID is pressing.

Promoting sexual and RH rights for women who use drugs necessitates considering the strong impact of gender-based violence on this population. In a study of 147 women in methadone treatment, 30.5% reported physical or sexual intimate partner violence in the year prior to the study [17]. A second study of 416 women in methadone treatment found that 46% had experienced physical or sexual intimate partner violence at baseline [18]. The potential for increased stigma and barriers to care created by the intersections of gender, gender identity, injection drug use, and violence necessitates a trauma-informed approach to sexual and RH care.

Incorporating patient preferences into medical service delivery may improve patient uptake of new services, and eliciting these preferences before implementation may ease the incorporation of a new clinical service line into existing practice [19–21]. The Consolidated Framework for Implementation Research (CFIR, Table 1) condenses constructs from 19 implementation models and spans 4 domains: intervention characteristics, outer setting, inner setting, and individual characteristics [23]. The CFIR has previously been applied to implementation research performed in studies of substance use disorder (SUD) treatments [24]. Compared to other theories such as Rogers’s Diffusion of Innovation, the CFIR is more comprehensive and emphasizes the necessity of prioritizing patient needs and resources during implementation design [24]. Given the comprehensive nature of the CFIR and its previous application to SUD research, we used it as our guide to implement RH services at an NSP in Seattle, WA. This NSP’s policy is to exchange needles in a 1:1 ratio. Hereafter, we refer to it as a syringe exchange program (SEP) to distinguish it from NSPs that do not have an exchange ratio. Our aim was to implement patient-centered RH care services at the SEP. Our objectives were to interview staff and clients to glean the barriers and facilitators to implementation; to utilize this information to inform the implementation; and to evaluate the implementation via chart audits, client surveys, and staff surveys.

**Table 1:** Consolidated Framework for Implementation Research (CFIR) constructs and descriptions [22]

Construct		Short description
I. Intervention characteristics		
A	Intervention source	Perception of key stakeholders about whether the intervention is externally or internally developed.
B	Evidence strength & quality	Stakeholders’ perceptions of the quality and validity of evidence supporting the belief that the intervention will have desired outcomes.
C	Relative advantage	Stakeholders’ perception of the advantage of implementing the intervention versus an alternative solution.
D	Adaptability	The degree to which an intervention can be adapted, tailored, refined, or reinvented to meet local needs.
E	Trialability	The ability to test the intervention on a small scale in the organization, and to be able to reverse course (undo implementation) if warranted.
F	Complexity	Perceived difficulty of implementation, reflected by duration, scope, radicalness, disruptiveness, centrality, and intricacy and number of steps required to implement.
G	Design quality & packaging	Perceived excellence in how the intervention is bundled, presented, and assembled.
H	Cost	Costs of the intervention and costs associated with implementing the intervention including investment, supply, and opportunity costs.
II. Outer setting		
A	Patient needs & resources	The extent to which patient needs, as well as barriers and facilitators to meet those needs, are accurately known and prioritized by the organization.
B	Cosmopolitanism	The degree to which an organization is networked with other external organizations.
C	Peer pressure	Mimetic or competitive pressure to implement an intervention; typically because most or other key peer or competing organizations have already implemented or are in a bid for a competitive edge.
D	External policy & incentives	A broad construct that includes external strategies to spread interventions, including policy and regulations (governmental or other central entity), external mandates, recommendations and guidelines, pay-for-performance, collaboratives, and public or benchmark reporting.
III. Inner setting		
A	Structural characteristics	The social architecture, age, maturity, and size of an organization.
B	Networks & communications	The nature and quality of webs of social networks and the nature and quality of formal and informal communications within an organization.
C	Culture	Norms, values, and basic assumptions of a given organization.
D	Implementation climate	The absorptive capacity for change, shared receptivity of involved individuals to an intervention, and the extent to which the use of that intervention will be rewarded, supported, and expected within their organization.
1	Tension for change	The degree to which stakeholders perceive the current situation as intolerable or needing change.
2	Compatibility	The degree of tangible fit between meaning and values attached to the intervention by involved individuals, how those align with individuals’ own norms, values, and perceived risks and needs, and how the intervention fits with existing workflows and systems.
3	Relative priority	Individuals’ shared perception of the importance of the implementation within the organization.
4	Organizational incentives & rewards	Extrinsic incentives such as goal-sharing awards, performance reviews, promotions, and raises in salary, and less tangible incentives such as increased stature or respect.
5	Goals and feedback	The degree to which goals are clearly communicated, acted upon, and fed back to staff, and alignment of that feedback with goals.
6	Learning climate	A climate in which: a) leaders express their own fallibility and need for team members’ assistance and input; b) team members feel that they are essential, valued, and knowledgeable partners in the change process; c) individuals feel psychologically safe to try new methods; and d) there is sufficient time and space for reflective thinking and evaluation.
E	Readiness for implementation	Tangible and immediate indicators of organizational commitment to its decision to implement an intervention.
1	Leadership engagement	Commitment, involvement, and accountability of leaders and managers with the implementation.
2	Available resources	The level of resources dedicated for implementation and on-going operations, including money, training, education, physical space, and time.
3	Access to knowledge & information	Ease of access to digestible information and knowledge about the intervention and how to incorporate it into work tasks.
IV. Characteristics of individuals		
A	Knowledge & beliefs about the intervention	Individuals’ attitudes toward and value placed on the intervention as well as familiarity with facts, truths, and principles related to the intervention.
B	Self-efficacy	Individual belief in their own capabilities to execute courses of action to achieve implementation goals.
C	Individual stage of change	Characterization of the phase an individual is in, as he or she progresses toward skilled, enthusiastic, and sustained use of the intervention.
D	Individual identification with organization	A broad construct related to how individuals perceive the organization, and their relationship and degree of commitment with that organization.
E	Other personal attributes	A broad construct to include other personal traits such as tolerance of ambiguity, intellectual ability, motivation, values, competence, capacity, and learning style.

## Methods

**Fig. 1:**
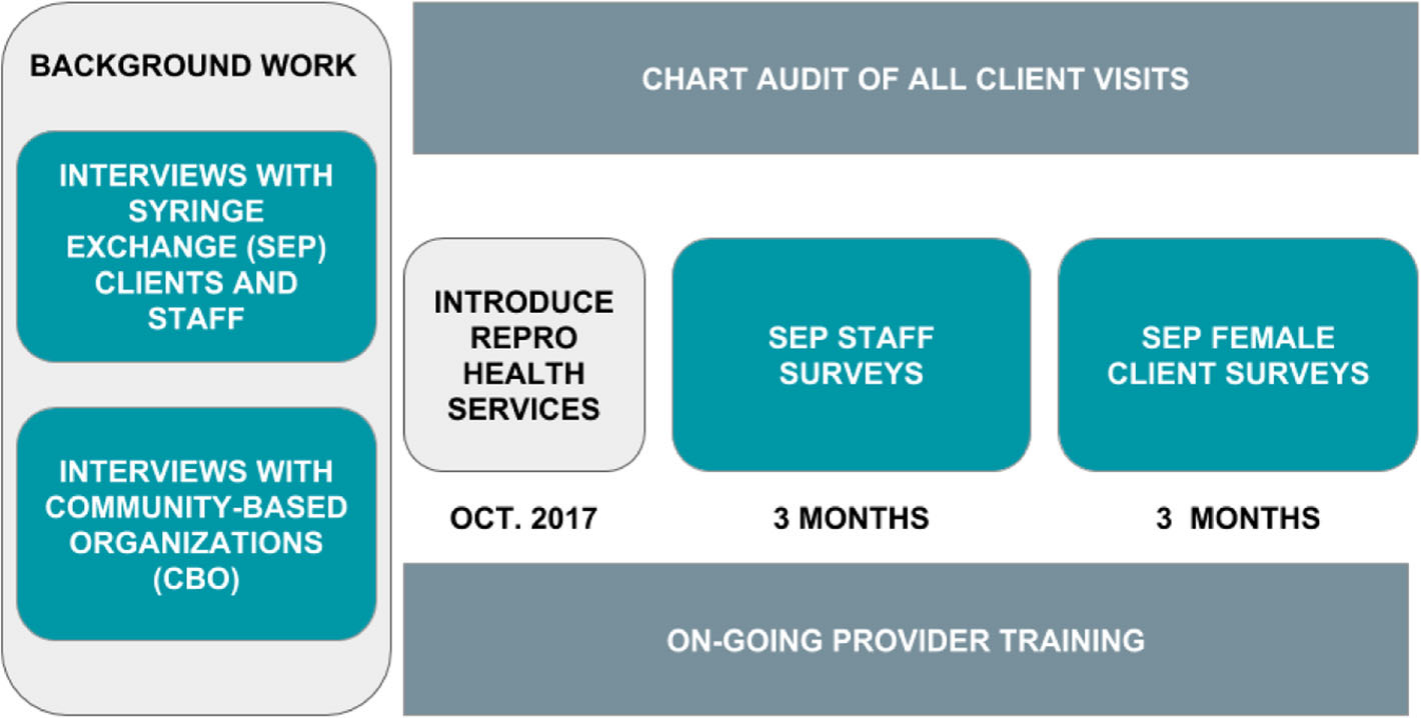
Diagram of project implementation phases and data collection

We used an iterative process to design, implement, and evaluate the provision of RH services within one Seattle-area SEP’s existing wound and primary care program. We used CFIR constructs to determine barriers and facilitators and patient preferences for RH service delivery before implementation. During implementation, we sought to determine patient and staff satisfaction with the new services, RH service uptake, and barriers and facilitators to continued service provision (Fig. 1).

This intervention occurred at an SEP in downtown Seattle, operated by Public Health Seattle and King County. The health department SEP consists of two sites (including the one involved in this study) and one mobile van. In 2016, the health department SEP exchanged over seven million syringes. At the study site, syringe exchange is provided 6 days per week and clinical services are provided on weekday afternoons. At the study site, during the 9-month pilot implementation, 280 total patients were seen, of which 111 were women.

First, we conducted qualitative interviews using the CFIR framework with 15 female clients, SEP staff, and staff at community-based organizations (CBOs) that provide social and health services for women who inject drugs (WWID). We interviewed 13 staff. Second, we designed a pilot program to introduce RH services at the SEP using major themes from the qualitative interviews. Finally, throughout the implementation, we surveyed SEP female clients and staff. We received 12 staff surveys at baseline and 7 surveys 3 months into the implementation. We collected 6 surveys from female clients who received RH care services during the implementation. We also conducted a chart audit of all healthcare services provided between October 2017 and June 2018 to assess RH service uptake. In particular, we reviewed charts of women obtaining clinical services to assess for adherence to Pap smear and STD screening guidelines, documentation of pregnancy intention, birth control utilization, and other well-woman services performed. The University of Washington Human Subjects Division approved the interview procedures as for human subjects research (University of Washington IRB number STUDY00001694) and declared that the surveys and chart audit were determined to be a quality improvement, not human subjects research (STUDY00003171). The Research Administrative Review Committee of Public Health Seattle King County approved the study as well.

### Collecting SEP staff and client feedback to inform RH service implementation

We used the CFIR to develop semi-structured interview guides for three key informant groups (SEP clients, SEP staff and clinicians, and CBOs serving WWID). We sought to identify barriers and facilitators to implementation, client and staff views on the intervention, and client-specific preferences for RH service delivery. We worked with SEP staff to define a purposive list of key internal and external stakeholders for interviews. Within the SEP, we interviewed at least one of each staff type (e.g., education specialist, clinician, social worker). We utilized snowball sampling and SEP staff’s knowledge of the network of organizations providing care to WWID to recruit external stakeholders. We conducted interviews from June 2017 to August 2017. Interviews lasted from 30 min to 1 h. All participants received a $10 gift card for participating; the incentive was at a level chosen to avoid coercion and in a format consistent with research previously performed at the SEP. Researchers experienced in interviewing techniques carried out the interviews, all of which were face-to-face. Interviews with clients and SEP staff were done in a private, closed room at the SEP. Interviews with external stakeholders were done at the locations of the interviewees’ choosing, generally in their offices. Interviews were audio-recorded and professionally transcribed, and transcripts were stored in a secure server. Recordings were erased after transcripts were uploaded to the server.

We recruited WWID at the SEP through fliers and by approaching female clients in person at the SEP. Female SEP clients were eligible to participate if they were ages 18–45, English-speaking, were assigned female at birth, were not menopausal, had never had a hysterectomy or sterilization procedure, and were not seeking pregnancy in the next year. For both staff and clients, written informed consent was obtained prior to each interview. Interview participants were assured of the deidentification of their data. Surveys were collected anonymously.

We used the CFIR as an a priori codebook and applied codes based on the definitions provided by CFIR. Using Dedoose (v 8.0.42), two independent coders coded line-by-line, applying CFIR codes. After coding the transcripts individually, coders met and reconciled coding discrepancies using a consensus process. Once all the interviews were coded, we used a thematic content analysis approach to determine major themes across and within key informant interview groups. We considered themes major if they occurred in at least two-thirds of transcripts. Major themes were presented to SEP staff and used to design the RH service delivery.

### Implementing RH services

We used major themes to design RH services, prioritizing the preferences of clients and perspectives of providers with experience serving WWID. Based on the literature on RH care provision for WWID reviewed during our research design, we also planned to focus on trauma-informed, low barrier care that incorporated the full range of reversible contraceptive options. Clinical services at the SEP have a complex infrastructure. Care providers are employees of an outside healthcare organization, but Public Health Seattle King County funds and distributes the SEP services themselves. The research team worked with both the SEP staff and clinicians to assess the feasibility of meeting clients’ RH service needs. Working collaboratively with these stakeholders, we agreed on a menu of services, a training program for SEP clinical providers and staff, advertising methods, and a date to launch RH services.

### Evaluating RH service provision

RH services were offered on Fridays from October 2017 to June 2018. We used two strategies to evaluate the introduction of RH services within the SEP clinical services: (1) SEP staff and clients were surveyed at 3 and 6 months into the implementation; (2) research staff conducted a chart audit to determine RH service uptake in the clinic.

#### SEP staff feedback surveys

We designed and administered a short survey using CFIR constructs to 10 SEP staff members (clinical and non-clinical) at 3 and 6 months post-implementation. We targeted our surveys to sample all the staff types at the SEP. The surveys focused on the fit of services within the SEP’s mission, the sustainability of services, client referrals to services, and staff comfort counseling women on a variety of RH topics. Questions included Likert scales, yes/no questions, and open-ended questions. Study staff gave paper survey copies to the SEP staff, who completed the surveys independently and returned them. Demographic information was not collected to preserve staff anonymity.

#### SEP client feedback surveys

We designed a survey based on Consumer Assessment of Healthcare Providers and Systems’ surveys on patient experience [25]. We offered this survey to clients who had received RH services. Study personnel stationed in the SEP offered the survey to all WWID who received RH care. This survey assessed patients’ experiences with their care and sought feedback to improve services. Questions were structured as Likert scales with one open-ended request for suggestions for service improvement. Surveys were anonymous. Study staff gave paper survey copies to the clients, who completed the surveys independently and returned them. Demographic information was not collected.

#### Chart audit

Once per week during the 9-month implementation, research staff reviewed electronic health data at the SEP on all days of clinical services, not just days where RH services were advertised. Research staff read through clinical notes to determine missed opportunities for RH service referrals, and to capture any RH service provision. We captured data from all female clients seen during the implementation time frame and abstracted whether pregnancy intention, birth control use, and Pap guidelines were discussed and documented. We also determined if clients received any STD testing or referrals for mammograms or other RH services beyond the capacity of the clinic. Given that the University of Washington Human Subjects Division viewed the implementation as quality improvement, not research, consent was not required for chart review. Charts were abstracted free of protected health information and data were stored in Research Electronic Data Capture (REDCap), a HIPAA-compliant web application.

## Results

### Collecting SEP Staff and client feedback to inform RH Service implementation

We completed 15 interviews with clients and 13 interviews with staff (from the Seattle SEP and other organizations serving WWID). All staff approached agreed to participate. Several clients declined to participate. The limiting factor for completing these interviews was availability of a private room. Thematic saturation occurred within these interviews.

All participating clients expressed favorable views toward integrating contraception at the SEP. Clients also expressed interest in expanded RH services at the SEP beyond contraception: STD testing, Pap smears, annual examinations, pregnancy testing, and pregnancy options counseling. In addition to these services, one client and one staff person mentioned abortion care; one client was interested in infertility care. Clients identified two main facilitators and two main barriers for RH service delivery. A walk-in care model and trusted providers trained in harm reduction were facilitators; male clinicians and long wait times were barriers. Living homeless, presence of judgmental staff, and need for pharmacy visits (i.e., medications not stocked onsite) were other barriers to accessing services.

Staff were enthusiastic about the prospect of providing RH services to clients and expanding healthcare service provision in general. Staff identified one main barrier and two main facilitators to implementing RH services at the SEP. The dearth of SEP service space and the competing demands on it (STD testing, social services) were the main barrier. Clients’ history of receiving poor care in clinical settings was a barrier one staff participant mentioned. Staff’s enthusiasm and interest in promoting the overall health of clients were the main facilitators.

Community-based organizations noted that traditional care settings embed barriers to care for their clients: stigma, fixed appointment times with potential penalties for missing appointments, providers not knowledgeable about injection drug use or harm reduction. They believed settings that eliminated stigma and prohibition, e.g., SEPs, would facilitate WWID’s access to care (Table 2).

**Table 2:** Characteristics of interviewees

Variable	*N* (%)
SEP clients (*n* = 15)	
Age median(IQR)	30 (23.5, 31)
Number of pregnancies in the past	
0	3 (20%)
1–2	4 (26.7%)
3–4	5 (33.3%)
4 or more	3 (20%)
Had sex with a man in the last 3 months	13 (86.7%)
Desires pregnancy in the next year	1 (6.7%)
Previous abortion	9 (60%)
Current form of contraception	
I do not use birth control	7 (46.7%)
Hormonal IUD	2 (13.3%)
Copper IUD	2 (13.3%)
Pill	1 (6.7%)
Shot	1 (6.7%)
Male condom	1 (6.7%)
Fertility Awareness Method	1 (6.7%)
CBO and SEP staff (*n* = 13)	
Years with organization median(IQR)	5.7 (3.1, 8.4)
What percentage of your time involves working with WWID? median(IQR)	30 (25, 45)
What is your role in your organization?	
Clinical	5 (38.5%)
Other client services	4 (30.8%)
Social work	2 (15.4%)
Management	2 (15.4%)

### Implementing RH services

We implemented RH services in October 2017. The primary investigator, an obstetrician-gynecologist, trained a female Advanced Registered Nurse Practitioner to provide evidence-based RH care services including STD testing, Pap smears, and contraceptive initiation and management of side effects and complications. The training occurred on-the-job through direct mentoring and discussion of high-yield RH topics. Services were offered on Friday afternoons. SEP staff and research staff advertised for services directly to female clients in the exchange and throughout the community via fliers and posting on community health education listservs.

Using feedback from clients and staff, we prioritized keeping a walk-in model of care for clients seeking RH services; focused our training on a female provider; trained all SEP staff in birth control options and pregnancy options counseling so they could provide some client education outside of the examination room; and offered a wide range of RH services including all forms of reversible contraception, STD testing, pregnancy testing, Pap smears, screening for urinary tract and vaginal infections, and mammogram referrals (Table 3).

**Table 3:** CFIR constructs and associated impacts on implementation*

CFIR construct [22]	Key informant group	Findings	Findings’ impact on implementation
I. Intervention characteristics (clients, SEP Staff, CBOs)			
Relative advantage	Clients	• SEP is already a convenient location to receive services • Clients want RH services offered alongside wound-care services • Clients wish to avoid pregnancy until they are ready to parent	• Reinforced decision to provide services at SEP • Need for contraception and pregnancy options services for clients and counseling skills for staff
	Staff	• Unmet need for all health care services in this population, including RH • SEP is a trusted, safe place where people can enter without judgement • Desire to test expanding clinical services at SEP	• Motivated expansion of implementation beyond contraception to fuller RH services
	CBO	• Separate preventive visits are challenging for clients to attend, even with advocates or case managers	• Reinforced integration of RH services into primary/wound care services
Design quality & packaging	Clients	• Services should include contraception and well-woman care • Services should be offered on a walk-in basis with short wait times to be seen • Clients prefer a female provider trained in harm reduction/trauma-informed care • Site should be able to dispense Rx at time of appointment	• Focused training efforts on female provider • Offered several contraceptive methods on-site • Maintained walk-in model of care
	Staff	• Walk-in services • Focus on novel ways to advertise so clients become aware of services, e.g. use SEP peer-educators to advertise services. • Collect many forms of contact information for test follow-up, and give clients option to walk in for test results. • Provide prenatal care, contraception, well woman care.	• Advertised services via flyers, bulletin board in SEP, word of mouth from staff and volunteers • Utilized walk-in model for follow-up and results as well as care
	CBO	• Trauma-informed and harm reduction training for all providers involved in delivering care. • Walk-in services • Ability to provide same-day contraception, examinations, and testing. • Avoid stigmatizing women’s desire to be pregnant or parent	• Emphasized trauma-informed approach in clinical training • Pregnancy options counseling training for staff with emphasis on harm reduction
Cost	Staff	• Concern over funding to pay for extra providers’ time • Matching funding source with program mission (i.e. broader healthcare fund rather than STD/HIV prevention)	• Train current providers and provide ongoing mentorship using trainers’ research time
II. Outer setting (clients, SEP Staff, and CBOs)			
Patient needs & resources	Clients	• Desired services: STD testing, contraception, pregnancy care, annual examinations, Pap smears	• Expansion from contraception to general RH care
	CBO	• Challenging to follow up with patients • Navigating consent with patients in the setting of active substance use and mental health diagnoses can be challenging	• Obtain multiple methods of contact, utilize non-traditional methods if client approves (e.g. leaving message for patient at shelter or day center) • Abstinence from substances is not a prerequisite for care or procedures; ability to express understanding for and desire for care/procedure is necessary
	Staff	• Clients need to develop relationships with staff in order to trust them • Pregnant clients are particularly likely to face judgment and barriers to care • Clients who do sex work need contraceptive methods other than condoms as condomless sex pays more • Contraceptive methods requiring daily or weekly user involvement are challenging • Living homeless and/or with substance use disorder means surviving takes up much of clients’ time, leaving less for preventive care	• Project staff spent weekly time assisting with syringe exchange to become familiar with clients • Acknowledge and combat the layered stigma of gender, pregnancy, and substance usage • Offer long-acting reversible contraception on-site • Make preventive services available where clients are seeking other services related to substance use disorder or living homeless
Peer pressure	CBO	• Few organizations work in the intersection of RH and substance use disorders • The nearest clinic has limited walk-in spots that may require an hours-long wait	• Reinforced need for integrating RH into SEP • Despite proximity of other clinics, lack of walk-in care is a barrier
	Staff	• Failure to treat patients’ substance use disorder with medication while inpatient frequently leads to adverse experiences and leaving against medical advice	• Emphasis on patient-centered care and therapeutic relationships
	Staff	• SEP cannot advertise any of its services on the sidewalk or outside of its building	• Unable to place poster or outward-facing advertisements for services
III. Inner setting (SEP staff)			
Structural characteristics	Staff	• Most staff are comfortable making referrals within and outside the organization	• Planned staff education around RH topics and created referral list for RH care
Networks & communications	Staff	• Management is open to suggestions from staff	• Fully involve all types of staff in formative work and evaluation
Culture	Staff	• Harm reduction and relationship building with clients are highly valued	• Create low barrier, friendly services
Implementation climate—tension for change	Staff	• Client needs and staff’s perceptions of needed improvements drive change	• Harness staff’s interest in implementing services given client demand
Readiness for implementation— leadership engagement	Staff	• SEP manager highly engaged with staff and responsive to feedback	• Harness manager’s energy and interest in promoting implementation
Readiness for implementation—available resources	Staff	• Space is limited • Examination room has footrests for gynecologic examinations • Highly functional electronic medical record available	• Limit RH-specific equipment to avoid straining limited space
IV. Characteristics of individuals (SEP staff)			
Knowledge & beliefs about the intervention	Staff	• Aware of increased effectiveness and lower user-related failure associated with IUDs and contraceptive implants • Desire for improved referral system for pregnancy options	• Designed referral brochure and educated staff on pregnancy options including abortion, adoption, parenting
Self-efficacy	Staff	• Very comfortable suggesting improvements and advocating for clients	• Utilized staff feedback in improving implementation

*Constructs without participant input or not impacting implementation are excluded from this table

We provided RH care services as planned (Table 4). We purchased long-acting methods (IUDs and implants) and kept them on-site. We sent shorter-acting contraceptive prescriptions to the pharmacy of the patient’s choice. When patients had positive test results (several STDs and a urinary tract infection were diagnosed), they most commonly followed up at the SEP to receive their positive results. Although patients seeking RH care were almost exclusively cisgender women, we did diagnose a cisgender male with urethritis and refer him for treatment.

**Table 4:** Chart audit of RH indicators during implementation (October 2017–June 2018)

Variable	*N* (%)
Total visits	587
Visits with female clients	182 (31% of all visits)
Female clients under 50	146 (80% of visits by women)
Primary complaint: repro health	22 (12.8%)
Primary complaint: wound care	116 (67.4%)
Primary complaint: primary care	59 (34.3%)
Pap Smear performed	8*
Patient up to date on Pap at end of visit	14 (8.1%)
STD testing performed	11*
Birth control options discussed	15*
Women reporting birth control use	24*
Birth control methods prescribed or placed on-site	5*
Mammogram referrals	4*

*% not given as may not have been indicated for all clients

### Evaluating RH service provision

#### SEP staff feedback surveys

We collected staff feedback on the implementation at 3 and 6 months from the initiation of services. Social work, management, clinical, and education specialists were surveyed. We received 10 surveys at 3 months and 6 surveys at 6 months (60% response rate at 6 months). Given the small number of staff surveys, the analysis involved descriptive statistics only. Staff felt strongly that the RH services fit into the SEP’s mission without disrupting other services. The majority of staff had referred clients to the SEP’s clinicians for RH services. Staff noted that having the services available more than 1 day per week would be ideal. They also noted that given the high burden of trauma and competing demands on women’s time, RH care services may not be women’s top priority. Staff also noted that even among women desiring services, inadequate advertising of services might hamper their uptake. Staff desired further training of all clinical providers to optimize sustainability.

#### SEP client feedback surveys

We collected client feedback on their experience with RH services. Although we had planned on collecting a group of 10 surveys at both 3 and 6 months from initiation of services, due to difficulty collecting these surveys, we collected surveys continually and received 6 surveys between months 3 and 6 of the implementation. Given the small number of surveys, the analysis was descriptive only. Overall, women reported positive experiences with clinicians. They felt providers explained clearly, listened carefully, showed respect, and gave easy to understand instructions. One person suggested having snacks available to improve the experience, and another suggested repositioning items within the examination room and having rapid testing available.

#### Chart audit

We reviewed the charts of patients seen at the SEP clinic during our implementation in order to characterize the clinic population and document the RH services provided. Over the period from 10/2017–6/2018, there were 587 visits to the clinic. Of these, 182 were women and 146 were women under 50 years old. The majority of clients sought wound care. As a result of the implementation, clients received Pap smears, STD screening, contraceptive counseling and provision, and referrals for mammography. These services were not available prior to the implementation.

## Discussion

By involving clients and staff in the planning and evaluation of this implementation, we aimed to optimize service acceptability and sustainability. We emphasized trauma-informed care to avoid re-traumatizing patients seeking care with us. Extending the SEP’s harm reduction model to our care meant occasionally seeing patients for conversation only without examinations. Thus, the clinical indicators in Table 4 do not account for all the patient interactions related to RH. For some women, simply having a positive experience with a gynecologist, even without an examination, was a new and valuable experience.

We noted strong demand from clients and strong support from staff during our formative qualitative work. However, the strong demand from clients did not translate into high uptake of services. This could have been secondary to competing priorities on women’s time, previous adverse experiences with RH care that discouraged women, lack of provider availability on days women sought services, and past sexual trauma leading to women’s avoidance of RH services.

RH service implementation has previously been described in the adolescent health literature in the context of a teen pregnancy prevention program. A study of 48 health centers in 10 communities found that increased implementation of evidence-based RH practices was associated with support from health center leadership, communication between leadership and staff, staff attitudes and beliefs [26]. Challenges in billing and coding for services and the absence of the aforementioned facilitators were the barriers noted. However, this study only drew its barriers and facilitators from staff and leaders of the centers, not from clients. Moore reported on the feasibility of combining contraception with a mobile NSP in Baltimore that targeted exotic dancers [8]. However, this report did not encompass barriers and facilitators to this service implementation.

A recent systematic review found that few studies using the CFIR elicited patient needs in regard to new service design and implementation, focusing instead on the staff, providers, and institutional units in which the implementation takes place. They also found that most studies only collected data from stakeholders during or post-implementation, missing an opportunity to identify facilitators and barriers before implementation began [27]. One formative evaluation using the CFIR found that interviewing both staff, providers, and patients before implementing an electronic pharmacist-led blood pressure monitoring intervention yielded important information about facilitators and barriers that could be addressed before implementation [28]. To our knowledge, there are no other implementation science projects that sought the input of PWID as consumers and stakeholders in their care.

Taking a wider view of WWID and the SEP, many WWID may be hesitant to utilize the SEP (and thus services contained in it) given the criminalization of their drug use. There are almost certainly barriers to receiving RH care that we did not elicit in our one-on-one interviews. Participants may have been reluctant to describe barriers to researchers in the one-on-one interviews with study staff. Focus group discussions, perhaps moderated by WWID, may have been useful to elicit more candid discussion. Our implementation provided services 1 day per week. Increasing the days on which a trained provider is available would improve access for women. Because the day of services was dictated by the availability of the female clinician, we did not inquire about a preferred day of services in our client interviews.

Despite the low uptake of services, we demonstrated the simplicity of adding RH services. To offer oral contraception, one only needs to measure blood pressure and screen for contraindications [29]. Injectable contraception can be initiated without blood pressure monitoring. Offering contraceptive implants also does not require a pelvic examination; however, it does require that providers receive training in insertion and removal from trainers vetted by the implant’s manufacturer [29, 30]. Providers who work at syringe exchanges and are accustomed to incision and drainage procedures will likely be comfortable with insertion and uncomplicated removals. Moreover, the instruments and supplies needed for wound care overlap with those needed for implant insertion and removal. The ability to collect and send a urine sample means the potential for pregnancy, STD, and urinary tract infection testing.

The main limitation of our work was our having trained only one provider at the SEP. We offered training to the SEP’s other providers; however, one declined and the other changed jobs during the course of the implementation. To improve sustainability, ideally, all the SEP’s clinicians would be comfortable providing RH care services. In our setting, all the clinicians had a background in primary care, making RH care within their scope of practice. Still, providing IUDs and contraceptive implants requires additional training. We offered one-on-one mentoring for the duration of the implementation. This mentorship was available post-implementation but did not continue secondary to competing clinical demands and staff’s other work-related duties.

A second limitation of our work is the small amount of feedback received from clients who received RH care services. Using study staff to collect these surveys may have suppressed negative feedback from clients dissatisfied with services. We took care to consider SEP clients’ needs when designing our services, but the paucity of feedback from clients having received the services limited our ability to continually improve them. In the future, involving WWID in interviews (e.g., having trained peer clients interview clients who utilized services) and utilizing more open-ended questions could yield more detailed and honest feedback. We would still face the logistical difficulty of imposing a further time burden on WWID who have already spent significant time at the SEP.

Finally, although the CFIR has previously been used in substance use research, we recognize that the criminalization, prohibition, and stigmatization of substance use are strong barriers in the outer setting that were not elicited in our interviews. These external pressures are likely to overshadow individual characteristics favoring implementation such as self-efficacy, advanced stage of change, and other personal attributes favoring implementation.

## Conclusions

Our work demonstrated that WWID utilizing the SEP had an interest in and unmet need for RH services. Moreover, implementing these services was feasible and acceptable to staff. We encourage others working with WWID to consider evaluating their clients’ RH needs and implementing RH care to meet them. Even the equipment or training to perform pelvic examinations, providers can offer services that clients may be unwilling or unable to accept elsewhere.

## Data Availability

Please contact the corresponding author for data requests.

## Abbreviations

CBO: Community-based organization
CFIR: Consolidated Framework for Implementation Research
IUD: Intrauterine device
NSP: Needle syringe program
PWID: People who inject drugs
RH: Reproductive health
SEP: Syringe exchange program
STD: Sexually transmitted disease
WWID: Women who inject drugs
